# Impact of halide variation on the optoelectronic properties of double perovskites

**DOI:** 10.1038/s41598-025-98686-6

**Published:** 2025-09-29

**Authors:** Deepak Choudhary, Mandeep Kaur, Govind Sharma, Rahul Palsaniya, Swarnkesh Loyalka, Satpal Singh, Updesh Verma, Prashant Yadav, Manendra

**Affiliations:** 1 Department of Physics, Chaudhary Charan Singh University, Meerut, 250001 UP India; 2 Department of Physics, Hariom Saraswati P.G. College Dhanauri, Haridwar, 247667 Uttarakhand India; 3 Department of Physics, Central University of Rajasthan, Bandarsindri, Ajmer, 305817 Rajasthan India; 4 Department of Physics, Rajiv Gandhi Govt. P.G. College, Mandsaur, 458001 Madhya Pradesh India; 5 Department of Physics, University of Rajasthan, Jaipur, 302004 Rajasthan India; 6 Department of Physics, Government College, Sumerpur, Pali, 306902 Rajasthan India; 7 Department of Physics, Banasthali Vidyapith, Banasthali, 304022 Rajasthan India; 8 Department of Physics, S.D. Government College, Beawar, 305901 Rajasthan India; 9 Department of Physics, Janta Vedic College, Baraut, Baghpat, 250611 UP India; 10 Department of Physics, Mahaveer University, Meerut, 250341 UP India; 11 Department of Physics & Astrophysics , University of Delhi, Delhi, India

**Keywords:** Double perovskite single crystals, Crystal growth, Sustainable optoelectronic technologies, Condensed-matter physics, Condensed-matter physics

## Abstract

Halide double perovskites of $A_{2}B(I)B(III)X_{6}$ (A = Cs, B(I) = Ag, B(III) = Bi, and X = Cl, Br) have gained a lot of attention as an alternative to lead perovskites due to their similar defect tolerance, low toxicity, high stability, high absorption coefficients, long carrier diffusion lengths, and tunable bandgaps. In this study, we used a slow-cooling method to synthesize single crystals of lead-free double perovskites, specifically cesium silver bismuth bromide ($Cs_{2}AgBiBr_{6}$) and cesium silver bismuth chloride ($Cs_{2}AgBiCl_{6}$), and investigated the impact of halide variations on the structural, electronics, and optical properties of these materials. According to X-ray diffraction (XRD), both materials crystallize in a cubic structure. In both compounds, the $[BiX_6]^{3-}$ and $[AgX_6]^{5-}$ octahedra (where $X = Br$ or *Cl*) were alternately connected. X-ray photoelectron spectroscopy (XPS) provided detailed insights into the electronic structure, showing slight variations in binding energies due to halide substitution. DFT calculations confirmed the stability of the cubic structure ($Fm\bar{3}m$) and revealed that the materials have an indirect band gap. A detailed investigation of the optical characteristics was carried out, with a focus on essential parameters such as the dielectric function, refractive index, absorption coefficient, and optical conductivity. These findings provide important insight into how the halide composition influences the optoelectronic properties of lead-free double perovskites. This understanding opens up new opportunities for green energy and substantially supports the ongoing advancement of high-efficiency and environment-friendly photovoltaic materials.

## Introduction

The metal halide perovskites have gained a lot of attention over the past decade which can be seen in the rapid enhancement of power conversion efficiency, now exceeding 25%^1^.Despite the remarkable efficiency and optoelectronic properties of lead-halide perovskites, the toxicity of lead (Pb) and the poor stability under environmental conditions such as heat, oxygen, moisture, electric field, and light are considered to be major barriers hindering its commercialization^2–5^. Overcoming these issues, many efforts have been made to increase the stability of perovskite materials and create lead-free alternatives. In order to develop alternatives to lead-halide perovskites, a cation substitution method has been investigated. This method alternatives two $Pb^{2+}$ cations with heterovalent cations, including monovalent B(I) and trivalent B(III) and provides lead-free halide double perovskites with the general formula $A_{2}B(I)B(III)X_{6}$^6–8^. Among these double perovskites, the all-inorganic $Cs_{2}AgBiX_{6}$ compounds (where X=Cl,Br) have attracted a lot of attention due to low toxicity and high stability. The substitution of $Ag^{+}$ and $Bi^{3+}$ for $Pb^{2+}$ provides an effective balance, retaining favorable electronic characteristics while reducing toxicity issues^9–11^.While finding the alternative of the lead-halide perovskite, it was thought that two $Pb^{2+}$ cations were replaced with the heterovalent (i.e., monovalent (I) and trivalent (III)) cations. This cation transmutation approach leads to the formation of lead-free halide double perovskites $A_{2}B(I)B(III)X_{6}$ structure^6–8^. In these double perovskites, all inorganic $Cs_{2}AgBiCl_{6}$ (X= Cl, Br) have attracted attention because of their high stability and low toxicity. The substitution of lead ($Pb^{2+}$) with silver ($Ag^{+}$) and bismuth ($Bi^{3+}$) offers a balanced approach in maintaining desirable electronic properties while addressing toxicity issues^9–11^. Halide anions ($Br^{-}$ and $Cl^{-}$) have a significant impact on the band gap of material, electronics structure, and stability, making halide variation a viable technique for enhancing performance. Members of this family, $Cs_{2}AgBiBr_{6}$ and $Cs_{2}AgBiCl_{6}$, provide a combination of structural stability, tunable band gaps, and various applications such as photovoltaics, catalysis, and detectors^12–15^. Tailor et al. investigated the carrier dynamics and polaronic features in $Cs_{2}AgBiBr_{6}$ crystals using temperature-dependent impedance spectroscopy and transient absorption spectroscopy^10,16^. Tailor et al. and the research groups also studied $Cs_{2}AgBiCl_{6}$ crystals for X-ray detection applications and demonstrated the imaging capabilities of this class of materials^17–19^. These materials are also useful for thermometric applications^20–22^. Bismuth (Bi) and silver (Ag) are important components of these materials, which contribute to highly efficient charge transport and high absorption coefficients^23–26^. The choice of halide ions significantly influences the crystal structure, optical, and electrical characteristics of $Cs_{2}AgBiX_{6}$ (X=Br,Cl), materials^27–30^. For example, a bromide-based material $Cs_{2}AgBiBr_{6}$ has a lower band gap ($\sim 2.1$ eV), making it suitable for photovoltaics and photodetectors. While chloride-based $Cs_{2}AgBiCl_{6}$ has a larger band gap ($\sim 2.6$ eV), it may be useful in ultraviolet (UV) detection, tandem solar cells, and other specialized photonic applications^17,31^. However, there are still challenges to understanding and improving the optoelectronic properties of these substances. In-depth investigations of the impact of halide variation are essential to improve their efficiency in real-world uses. The first-principles density functional theory (DFT) is a useful tool to investigate the structural, optical, and electrical properties of novel materials. DFT makes it possible to make accurate quantum-level predictions about the behavior of materials, providing deep insights into their fundamental properties. Presently, DFT-based investigation have provided useful details on the electronic band structures, optical properties, and stability of halide perovskites, supporting work to develop novel materials with optimum performance^32^.

In this study, single crystals ($Cs_{2}AgBiBr_{6}$ and $Cs_{2}AgBiCl_{6}$) are synthesized using a slow-cooling method and examined their structural and optical properties using X-ray diffraction (XRD), X-ray photoelectron spectroscopy (XPS), Raman spectroscopy, and UV spectroscopy. These findings are validated by applying first-principles DFT calculations to the structural, electrical, and optical properties of $Cs_{2}AgBiBr_{6}$ and $Cs_{2}AgBiCl_{6}$ within the framework of Generalized Gradient Approximation with Perdew-Burke-Ernzerhof Functional (GGA-PBE), as well as Tran-Blaha modified Becke-Johnson (TB-mBJ). The purpose of this investigation is to examine how variations in halide composition affect structural stability, electronics, and optical properties. This combined study will provide insight into these promising compounds $Cs_{2}AgBiBr_{6}$ and $Cs_{2}AgBiCl_{6}$ for the development of high-efficiency optoelectronic devices. Additionally, these findings contribute to the development of sustainable and economically viable lead-free perovskites.

## Experimental and computational methodology

### Materials

Silver (I) chloride (AgCl, 99.99 %), bismuth (III) chloride ($BiCl_{3}$, 99 %), cesium (I) chloride (CsCl, 99 %), bismuth bromide ($BiBr_{3}$) ($> 98 \%$), silver bromide (AgBr, 99 %), cesium bromide (CsBr) (99.9 % trace metal base) were bought from Sigma Aldrich and hydrochloric acid (HCl 35-38 %) from s d fine-CHEM limited (SDFCL). Sisco Research Laboratories Pvt. Ltd. (SRL) supplied the hydrobromic acid (HBr, 48-49 % in water). Every chemical was utilized without any additional purification.

### Crystal growth

The $Cs_{2}AgBiBr_{6}$ crystal growth was started by first dissolving 2.0 mmol CsBr and 1.0 mmol $BiBr_{3}$ in HBr acid at room temperature. AgBr was then added and the solution was briefly stirred. The vial containing the prepared solution was heated to 130 $^{0}\text {C}$ and maintained at this temperature until the solution became clear and transparent. Subsequently, the vial was transferred to a closed heating oven programmed for controlled cooling. The temperature was initially held constant at 130 $^{0}\text {C}$ for 12 hours to ensure uniform conditions. Following this, the temperature was gradually reduced at a controlled rate of 0.3 $^{0}\text {C}$ per hour from 130 $^{0}\text {C}$ down to room temperature. This controlled cooling process typically requires approximately 8 days to complete and facilitates the growth of large-sized crystals. The same procedure was followed for the growth of $Cs_{2}AgBiCl_{6}$ crystals. In this process, 2.0 mmol of CsCl and 1.0 mmol of $BiCl_{3}$ were dissolved in HCl acid at room temperature, followed by the addition of AgCl.

### Characterizations

A DSLR camera was used to capture pictures of crystals. A Rigaku powder X-ray diffractometer with a Cu $K_{\alpha }$ radiation ($\lambda = 0.1542$nm) and a HyPix-3000 semiconductor detector was used to estimate the structure of the formed crystals. Using the diffused reflectance (DRS) mode on an Agilent Cary 100 UV-Vis spectrophotometer, ultraviolet-visible (UV-Vis) absorption spectra of the formed crystals were obtained. The absorption can be measured in a range of 200-800 nm.

### DFT calculations

The structure of halide perovskites $Cs_{2}AgBiX_{6}$ (X = Br, Cl) is investigated using density functional theory (DFT) techniques. The Wien2k simulation code^33^ uses a linearized augmented plane-wave basis set to solve the Kohn-Sham (KS) equations and determine ground-state charge densities. Based on this technique, Perdew, Burke, and Ernzerhof (PBE) parameterized a generalized gradient approximation (GGA) to approximate exchange-correlation potentials. To improve the accuracy of bandgap calculations, we add the modified Becke-Johnson (mBJ) exchange potential to GGA, which has a tendency to underestimate bandgaps^34^. In order to ensure convergence, the charge density and the total energy must be $10^{-4}$ eV and $10^{-4} R_{y}$, respectively. The unit cell is divided into muffin-tin spheres, which are assigned radii, with the interstitial regions accounted for in the calculation. Inside the muffin-tin spheres, the wavefunctions are expanded in terms of atomic-like orbitals up to an angular momentum quantum number of $l_{(max)}= 10$, while the plane-wave basis set for the interstitial space is limited by setting $R_{MT} K_{MAX} =7.0$, where $R_{MT}$ is the smallest muffin-tin radius and $K_{MAX}$ is the maximum value of the plane-wave vectors. The Brillouin zone (BZ) is sampled using 3000 k-points for accurate energy convergence. We also set the $G_{max}$ value to 12, ensuring a comprehensive analysis of diverse material properties. Additionally, the electronic and optical properties were rigorously evaluated using the selected computational frameworks and parameters. This methodological rigor provided a robust and detailed understanding of the halide perovskites $Cs_{2}AgBiX_{6}$ (X = Br, Cl).

## Results and discussions

### Crystal growth and structural properties

**Fig. 1:**
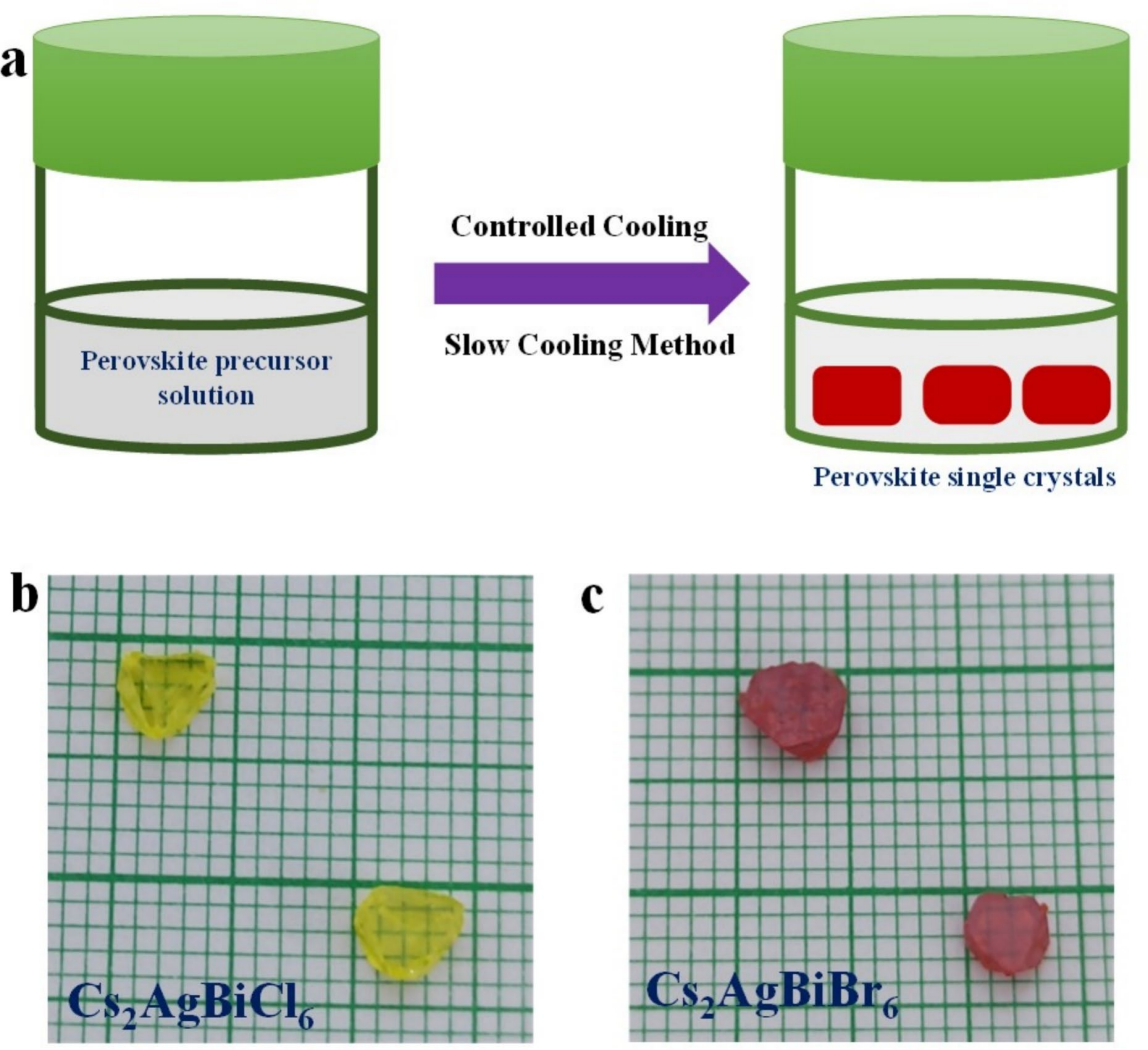
(**a**) Schematics of slow cooling method for crystal growth, and images of grown crystals: (**b**) $Cs_{2}AgBiCl_{6}$ and (**c**) $Cs_{2}AgBiBr_{6}$.

The single crystals of $Cs_{2}AgBiCl_{6}$ and $Cs_{2}AgBiBr_{6}$ are prepared via a slow cooling technique (Fig. 1a). This technique was based on the use of hydrochloric (HCl) and hydrobromic (HBr) acid to generate a hydrogel to help dissolve the precursory material. Details of this technique are available in the crystal growth section. Figure 1b ($Cs_{2}AgBiCl_{6}$) and Fig. 1c ($Cs_{2}AgBiBr_{6}$) show an image of the self-grown crystals of long side scale around a millimetre. Among them, their crystals are in a truncated form of octahedron. We also got a couple of small single ones apart from millimeter-size crystals. Using the generalized gradient approximation (GGA) predicts the structure of $Cs_{2}AgBiX_{6}$ (X = Br, Cl) which is investigated to stabilize in a cubic $Fm\bar{3}m$ structure, space group number (225). To validate this, we first computed the tolerance factor:

1 $$\begin{aligned} \tau _{X}=\frac{r_{Cs}+r_{X}}{\sqrt{2}\left( \frac{r_{Ag}+r_{Bi}}{2} +r_{X}\right) } \end{aligned}$$

The ionic radii where $r_{Cs}$, $r_{Ag}$, $r_{Bi}$ and $r_{X}$ ($X = Br, Cl$) are crucial parameters to determine the stability of the halide perovskite structure. The computed values of $(\tau _{X})$ for $Cs_{2}AgBiBr_{6}$ and $Cs_{2}AgBiCl_{6}$ can be found in Table1, confirming these compounds are most stable in the cubic structure.

**Table 1:** The determined values of a (Å) (lattice constant), V (volume), B (bulk-modulus), $E_{0}$ (ground state energy) , and $\tau _{X}$ (tolerance factor) of $Cs_{2}AgBiX_{6}$ (X = Br, Cl).

Compound	a (Å)	Volume $(a.u^{3})$	B(GPa)	$E_{0}(R_{y})$	$\tau _{X}$
$Cs_{2}AgBiBr_{6}$	11.44	2532.3588	40.85	-116239.4291	0.89
$Cs_{2}AgBiCl_{6}$	10.95	2216.5418	27.78	-90497.8705	0.90

**Fig. 2:**
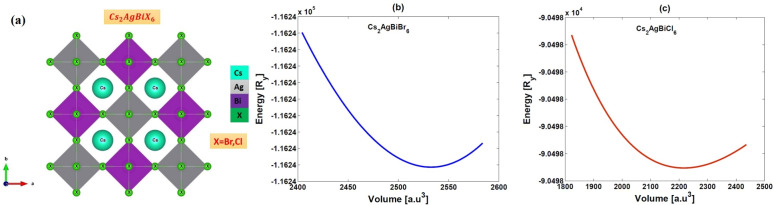
(**a**) The crystal structure representation of $Cs_{2}AgBiX_{6}$ (X = Br, Cl), and (**b**)-(**c**) Optimization plot of investigated double perovskites $Cs_{2}AgBiX_{6}$ (X = Br, Cl).

**Fig. 3:**
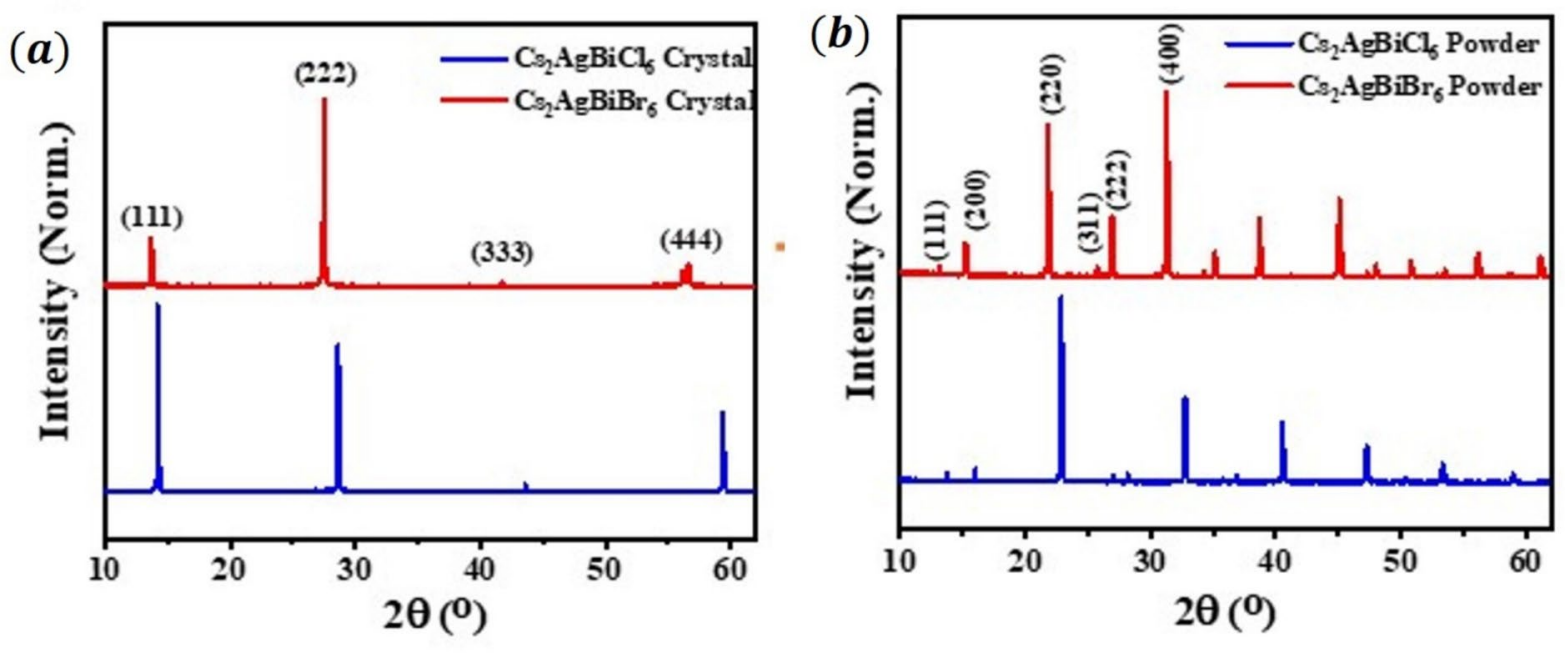
(**a**) XRD pattern of $Cs_{2}AgBiCl_{6}$ and $Cs_{2}AgBiBr_{6}$ crystal. (**b**) XRD pattern of $Cs_{2}AgBiCl_{6}$ and $Cs_{2}AgBiBr_{6}$ powder.

The crystal structure plays a vital role to determine their photophysical characteristics. It was proposed that these double perovskites have a three-dimensional framework of corner connected octahedra, with $Cs^{+}$ ions occupying the cuboctahedral cavities in the framework. The double perovskite structure consists of alternating $Ag^{+}$ and $Bi^{3+}$ centered octahedra in all three directions building up a superstructure that is typically referred to as rock salt ordering. A $Cs_{2}AgBiCl_{6}$ double perovskite is composed of a $Cs^{+}$ ion at the center of cuboctahedra with alternating $[BiCl_{6}]^{-3}$ and $[AgCl_{6}]^{-5}$ octahedral units (Fig. 2 a). The crystal structure of ordered $Cs_{2}AgBiBr_{6}$ is consist of corner-connected $[BiBr_{6}]^{-3}$ and $[AgBr_{6}]^{-5}$ octahedras (Fig. 2 a). However, the electronic dimensionality in these perovskites has been described as 0D because of the spatial isolation between the $[AgX_{6}]^{-5}$ and $[AgX_{6}]^{-3}$ octahedra. The 0D electronic dimensionality causes the localization of photoexcited charge carriers and results in the high charge-carrier effective masses. The stability of the $Fm\bar{3}m$ cubic structure is further confirmed through structural optimization. Structural stability is evaluated by plotting the total energy as a function of volume. The lowest energy point on this energy-volume (E-V) curve represents the most stable configuration of the material. Figure 2(b-c) illustrates the minimum in the E-V curve, indicating the ground-state energy of these materials. The results of structural optimization parameters such as lattice constant (a), bulk modulus (B), volume (V) and the minimum energy ($E_{0}$) are summarized in Table 1. The Birch-Murnaghan equation of state (EOS)^35^ is employed to derive the basic structural parameters from the total energy vs. total volume curve for the primitive unit cell.

**Fig. 4:**
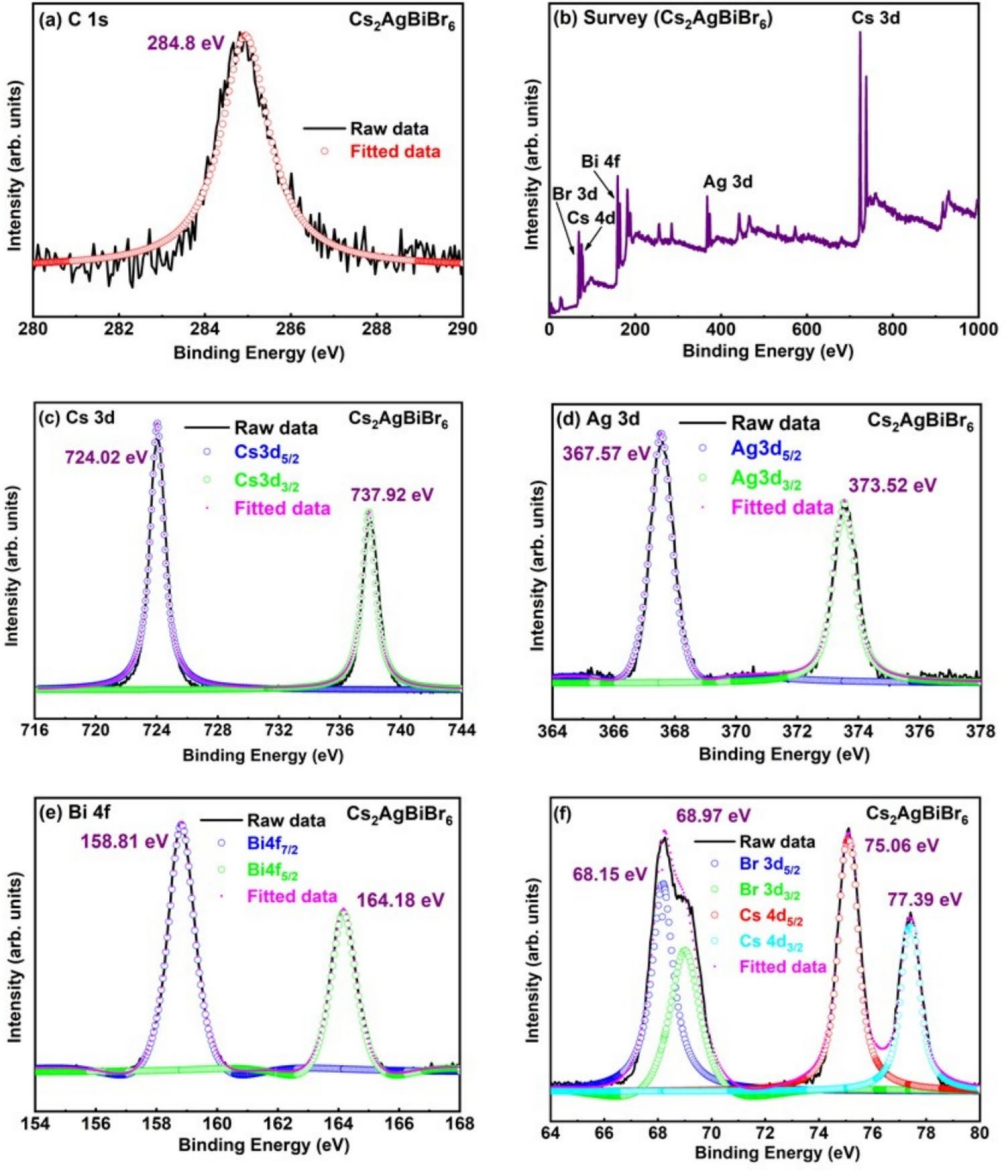
XPS spectra of (**a**) C 1s, (**b**) Survey scan, (**c**) Cs 3d, (**d**) Ag 3d, (**e**) Bi 4 f, (**f**) Br 3d core level obtained for $Cs_{2}AgBiBr_{6}$ sample.

**Fig. 5:**
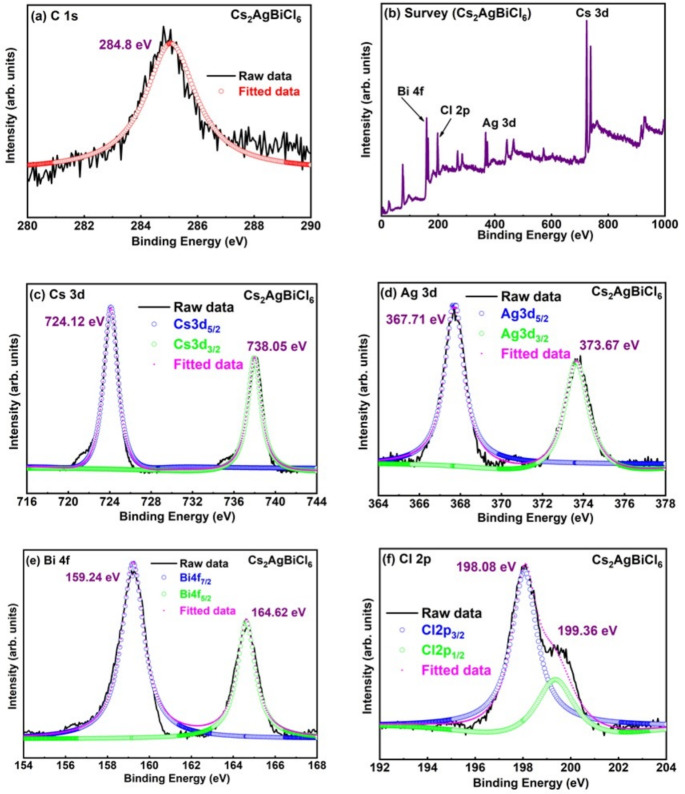
XPS spectra of (**a**) C 1s, (**b**) Survey scan, (**c**) Cs 3d, (**d**) Ag 3d, (**e**) Bi 4 f, (**f**) Cl 2p core level obtained for $Cs_{2}AgBiCl_{6}$ sample.

The X-ray diffraction (XRD) method was used to examine the crystalline quality of as-grown lead-free single crystals (Fig. 3). The $Cs_{2}AgBiCl_{6}$’s main peaks, according to the XRD pattern, are at $2\theta = 14.61^{\circ }$ (111), $29.12 ^{\circ }$ (222), $43.96 ^{\circ }$ (333), and $59.70 ^{\circ }$ (444) [Fig. 3(a) and 3(b)] and for the $Cs_{2}AgBiBr_{6}$, at $2\theta = 14.09^{\circ }$ (111), $27.78 ^{\circ }$ (222), $42.06 ^{\circ }$ (333), and $56.95 ^{\circ }$ (444) [Fig. 3(a) and 3(b)]. The $Cs_{2}AgBiBr_{6}$ standard pattern located in the database (ICSD collection # 252164)^36^ is in good agreement with the diffraction pattern that was obtained. This demonstrates that our solution growth methodology’s as-grown crystals are in the $Cs_{2}AgBiBr_{6}$ phase. From Cl ($r = 1.81 ({\text{\AA }})$) to Br ($r = 1.96({\text{\AA }})$), we see that all of the major peaks move towards a lower value, indicating that the spacing of the lattice rises as the anion size increases. According to crystallographic study, every synthesized sample is crystalline and has a distinctive cubic structure in the centrosymmetric $Fm\bar{3}m$ space group. Using $a = b = c = 10.77({\text{\AA }}), \alpha =\beta =\gamma = 90^{\circ }$ for $Cs_{2}AgBiCl_{6}$ crystal and $a = b = c = 11.28({\text{\AA }})$, with $\alpha =\beta =\gamma = 90^{\circ }$ for $Cs_{2}AgBiBr_{6}$ crystal, the lattice parameters are determined. $(AgBr_{6})^{5-}$ lattice vibrations predominate because the unit cell axis is roughly twice as large as the single $MAPbBr_{3}$ perovskites, and Ag-Br ($2.822(5)({\text{\AA }})$) has a little stronger bonding strength compared to Bi-Br ($2.813 (5)({\text{\AA }})$)^37^. The full width at half maximum (FWHM) is measured for all planes. FWHM is $0.048 ^{\circ }$ (111), $0.036^{\circ }$(222), $0.036^{\circ }$ (333), and $0.048^{\circ }$ (444). FWHM is $0.060^{\circ }$ (111), $0.024 ^{\circ }$ (222), $0.048 ^{\circ }$ (333), and $0.048 ^{\circ }$ (444), for the $Cs_{2}AgBiBr_{6}$ crystal. The exceptional crystalline quality associated with these crystals is suggested by their extremely distinct and sharp XRD peaks that have a small FWHM. One family of peaks in these fabricated single crystals strongly illustrates how single crystals grow.

### XPS analysis

XPS analysis was used to conduct quantitative investigations of the electronic structures and chemical characteristics of $Cs_{2}AgBiBr_{6}$, and $Cs_{2}AgBiCl_{6}$ samples. Figures 4 and 5 depict the results of XPS showing the change in binding energy with halide variation. The PsdVoigt2 peak function is used to fit the core level peaks of all the graphs. The C 1s spectrum signal was used to calibrate the binding energies of XPS spectra (Figs. 4a and 5a). The survey scan XPS spectra of samples are displayed in Figs. 4b and 5b, confirms the elemental composition of $Cs_{2}AgBiBr_{6}$ and $Cs_{2}AgBiCl_{6}$, respectively. The XPS results of C 1s, Survey scan, Ag 3d, Cs 3d, Br 3d and Bi 4 f core level were recorded for the $Cs_{2}AgBiBr_{6}$ sample. The C 1s peak located at 284.8 eV, is utilized to calibrate all of the spectra. The Cs element matches the Cs 3d doublet peaks of the $Cs_{2}AgBiBr_{6}$ sample, which are located at 724.02 eV (Cs $3d_{5/2}$) and 737.92 eV (Cs $3d_{3/2}$), as shown in Fig. 4c^38^. The Cs element is also matched through the Cs 3d doublet peaks in the $Cs_{2}AgBiCl_{6}$ sample in Fig. 5c, which are located at 724.12 eV (Cs $3d_{5/2}$) and 738.05 eV (Cs $3d_{3/2}$)^38^. The two peaks were separated by an energy value of 14 eV. Peaks at 367.57 eV (Ag $3d_{5/2}$) and 373.52 eV (Ag $3d_{3/2}$) in the Ag 3d XPS spectra of the $Cs_{2}AgBiBr_{6}$ sample indicate a monovalent oxidation state of Ag, which is displayed in Fig. 4d^38^. Similarly, Fig. 5d displays the Ag 3d XPS spectrum of the $Cs_{2}AgBiCl_{6}$ sample, which shows peaks at 367.71 eV (Ag $3d_{5/2}$) and 373.67 eV (Ag $3d_{3/2}$), which similarly correspond to a monovalent oxidation state of Ag^38^. There is a 6 eV energy difference between the two peaks. The Bi $4f_{7/2}$ and Bi $4f_{5/2}$ peaks were discovered at 158.81 eV and 164.18 eV, respectively, for the $Cs_{2}AgBiBr_{6}$ sample, as shown in Fig. 4e. Fig. 5. XPS spectra of (a) C 1s, (b) Survey scan, (c) Cs 3d, (d) Ag 3d, (e) Bi 4 f, (f) Cl 2p core level obtained for $Cs_{2}AgBiCl_{6}$ sample. Figure 5e depicts that the Bi $4f_{7/2}$ and Bi $4f_{5/2}$ peaks were found at 159.24 eV and 164.62 eV, respectively for $Cs_{2}AgBiCl_{6}$ sample. The two peaks were separated utilizing an energy value of 5 eV. In the $Cs_{2}AgBiBr_{6}$ sample, the Br 3d doublet peaks located at 68.15 eV and 68.97 eV are shown in Fig. 4f and are ascribed to Br $3d_{5/2}$ and Br $3d_{3/2}$, respectively. The two peaks have an energy difference of 0.82 eV. The Br XPS spectra also shows two additional peaks at higher binding energy side at around 75 eV and 77 eV due to the existence of Cs 4d core level^39^. The Cs4d peaks are observed in Figure 4f corresponding to $Cs^{1+}$ state^40^. The Cs 4d spectra shows two definite strong peaks at 75.06 eV and 77.39 eV corresponding to $4d_{5/2}$ and $4d_{3/2}$, respectively (Fig. 4f). The two peaks were separated using an energy value of 2.33 eV. The Cl 2p peak can be separated between two peaks that arise from Cl $2p_{3/2}$ and Cl $2p_{1/2}$, respectively, at binding energies of 198.08 eV and 199.36 eV (Fig. 5f) in the case of $Cs_{2}AgBiCl_{6}$ sample. The difference in energy between the two peaks is 1.28 eV. The peak of Cs 3d, Ag 3d and Bi 4f shifts 0.1 eV, 0.2 eV and 0.4 eV, respectively towards a higher binding energy when comparing the $Cs_{2}AgBiBr_{6}$ sample to the $Cs_{2}AgBiCl_{6}$ sample. The small variation in binding energy values depicts the small modification in electronic structure of both samples^41^ . Hence XPS results depicts the involvement of all the elements during synthesis process.

**Fig. 6:**
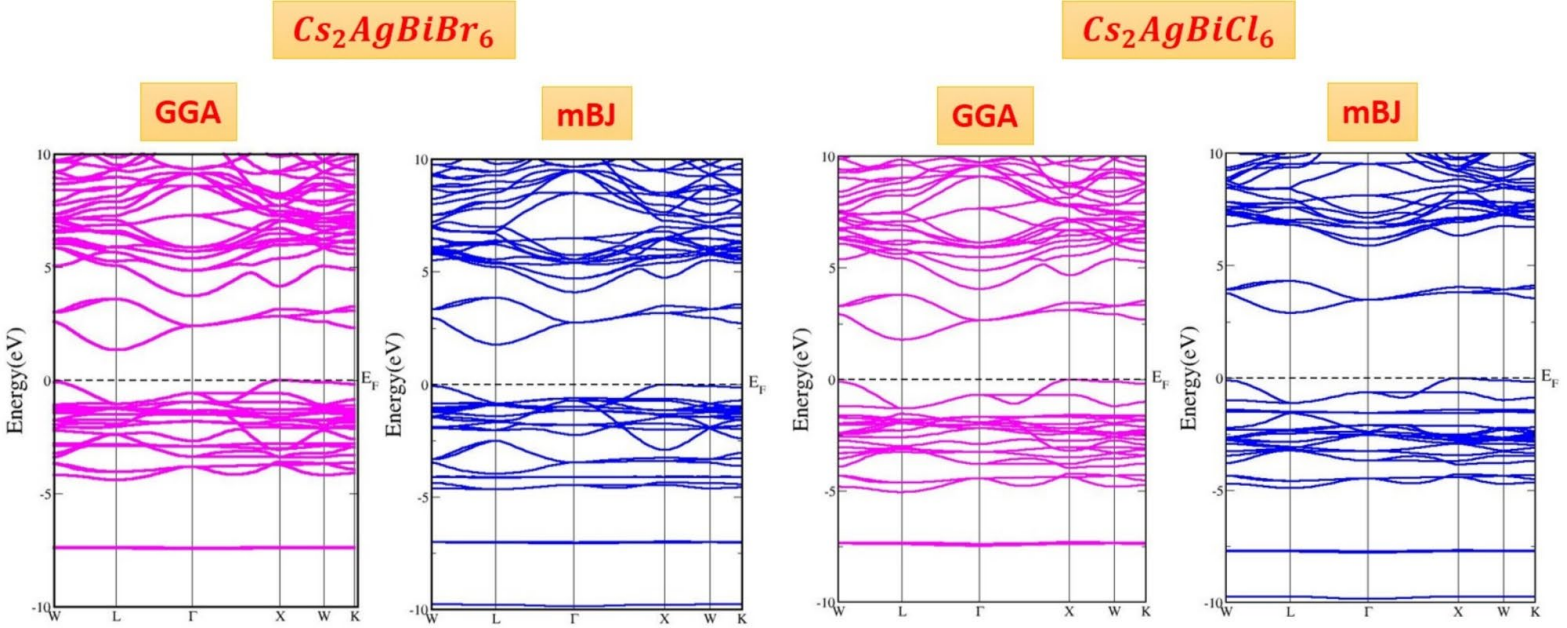
Generalized Gradients Approximation and GGA + mBJ band structure of $Cs_{2}AgBiX_{6}$ (X = Br, Cl).

**Fig. 7:**
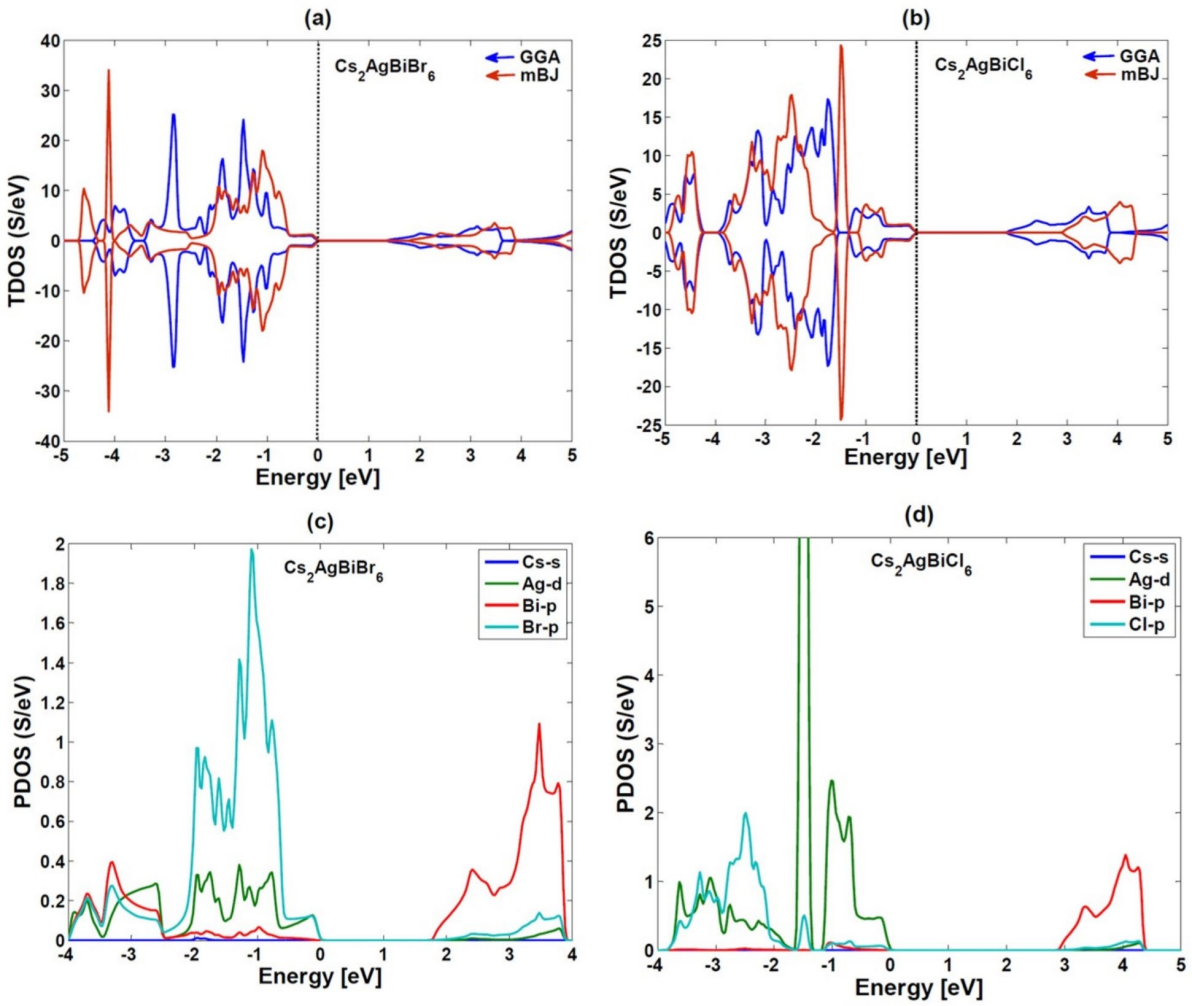
(**a**)-(**b**) Total density of states (TDOS) of compound using PBE-GGA and mBJ approach, and (**c**)-(**d**) Partial density of state (PDOS) of compound $Cs_{2}AgBiBr_{6}$ and $Cs_{2}AgBiCl_{6}$ respectively using mBJ approach.

### Electronic properties

The electronic characteristics of materials, such as total density of states (TDOS), partial density of states (PDOS), and band structure, are essential for understanding its behavior in various applications. These properties are obtained using Density Functional Theory (DFT), which is based on the principle that the characteristics of an interacting fermion system can be determined from its electron density, rather than the complex many-body wavefunction. However, the absence of a precise exchange-correlation function outside of the free-electron gas model presents a significant obstacle. To overcome this limitation, a variety of approximations have been developed, enabling the accurate computation of numerous physical properties. Despite these improvements, approximated exchange-correlation functionals are still an integral part of the calculations. SCF calculations were utilized in this work to determine the electronic band structure and band gap of the materials. These calculations employed potential functionals described in the computational section. Specifically, it is well recognized that GGA functionals, despite their frequent use in DFT, generally underpredict the electronic band gaps of semiconductors^42^. To overcome this limitation and improve the precision of the computed band gaps, we used the Tran-Blaha modified Becke-Johnson (TB-mBJ) potential. However, the TB-mBJ potential further refines the exchange-correlation potential by improving electron localization and offering a more realistic description of the electronic structure. With this modification, band gap calculations are more accurate as well as aligned with the experimental results. As a result, the TB-mBJ approach proves to be particularly effective for electronic structure calculations. Figure 6 illustrates the band structure curves for $Cs_{2}AgBiBr_{6}$ and $Cs_{2}AgBiCl_{6}$. These graphs provide significant details about the electronic behavior of the materials being studied by showing the relationship between the wave vector k and the energy within the first Brillouin zone.

**Table 2:** Estimated values of band gap and nature of these alloys in GGA and TB-mBJ methods.

Compound	Method	Bandgap [eV]	Nature	Band transition
$Cs_{2}AgBiBr_{6}$	GGA	1.35	Indirect	$L-X$
	mBJ	1.80	Indirect	$L-X$
	Experimental	2.02	Indirect	
$Cs_{2}AgBiCl_{6}$	GGA	1.55	Indirect	$L-X$
	mBJ	2.90	Indirect	$L-X$
	Experimental	2.50	Indirect	

Figures 6 and 7 show the electronic properties, total and partial densities of states (TDOS and PDOS) of $Cs_{2}AgBiBr_{6}$ and $Cs_{2}AgBiCl_{6}$, based on their estimated band structures.

The band structures are located along the Brillouin zone (BZ) symmetry line and have energies ranging from -10 eV to 10 eV. The electronic band profiles of $Cs_{2}AgBiX_{6}$ (X = Br, Cl) are investigated using both the PBE-GGA and TB-mBJ functionals, as shown in Fig. 6. The band structure Figures depict the energy levels as a function of momentum along the high-symmetry paths in the Brillouin zone $(W\rightarrow L\rightarrow \Gamma \rightarrow X \rightarrow W \rightarrow K)$. Table 2 summarizes the computed band gaps for each functional. In the Brillouin zone, estimated band structures indicate that the conduction band minimum (CBM) is located at the X point. In contrast, the valence band maximum (VBM) is located at the L point. These characteristics have significant effects on the potential application of materials in optoelectronics and are needed to understand electronic properties of materials. Figure 7 shows how the distribution of electronic states is affected by the contribution of different atomic orbitals from Cesium (Cs), Silver (Ag), Chlorine (Cl), and Bromine (Br). Our findings, the d-orbitals of the silver (Ag) atoms play an important role in determining the borders of the conduction and valence bands. These d-state bands have a high density of states at the Fermi level, indicating that electronic states play an important role in the conductivity of material. The orbitals of the Cs-s, Ag-d, Bi-p, and Br/Cl-p orbits show remarkable hybridization and modify the electronic structure with favorable energy ranges. These materials are potential candidates for optoelectronic applications due to the range of band gaps.

### Optical properties

The optical properties of material must be understood in order to assess its potential for cutting-edge technological applications such as photovoltaics and optoelectronic devices. The interaction between incoming electromagnetic radiation and the material, as well as its dependency on photon energy, are described in detail by the complex dielectric function, $\varepsilon (\omega )$. The mathematical expression for this theoretical framework, which was first presented by Ehrenreich and Cohen^43^, is as follows:

2 $$\begin{aligned} \varepsilon(\omega ) = \varepsilon _1(\omega ) + i\varepsilon _2(\omega ) \end{aligned}$$

Here the real component of the dielectric function, $\varepsilon_1(\omega )$, describes the material’s polarization response, whereas the imaginary part, $\varepsilon_2(\omega )$, is linked with light absorption^44^. $\omega$ represents the angular frequency of the incident electromagnetic radiation. The imaginary component, $\varepsilon_2(\omega )$, is derived using the following expression:

3 $$\begin{aligned} \varepsilon _{2}(\omega )=\frac{4\pi ^2 e^{2}}{ m^{2}\omega ^{2}}\int \sum _{i,j}|\left\langle i|M|j\right\rangle |^{2} Qd^{3}k \end{aligned}$$

Here, $Q=f\left( kn \right) \left[ 1- f\left( kn'\right) \right] \delta \times \left( E_{f}-E_{i}- \hbar \omega \right)$, e is the electron charge, m is the electron mass, $E_f$ and $E_i$ represent the final and initial electronic states, respectively, *f*(*kn*) is the Fermi-Dirac distribution, M denotes the transition matrix element, and $\delta \times \left( E_f-E_i- \hbar \omega \right) )$ ensures energy conservation. The real part of the dielectric function, $\varepsilon _{1}(\omega )$, is determined from $\varepsilon _{2}(\omega )$ using the Kramers-Kronig relation:

4 $$\begin{aligned} \varepsilon _{1}(\omega )=1+\frac{2}{\pi }\int _{0}^{\infty }\frac{\varepsilon _{1}\left( \omega ' \right) \omega '}{\left( \omega ^{'2}- \omega ^{2} \right) }d\omega ' \end{aligned}$$

**Fig. 8:**
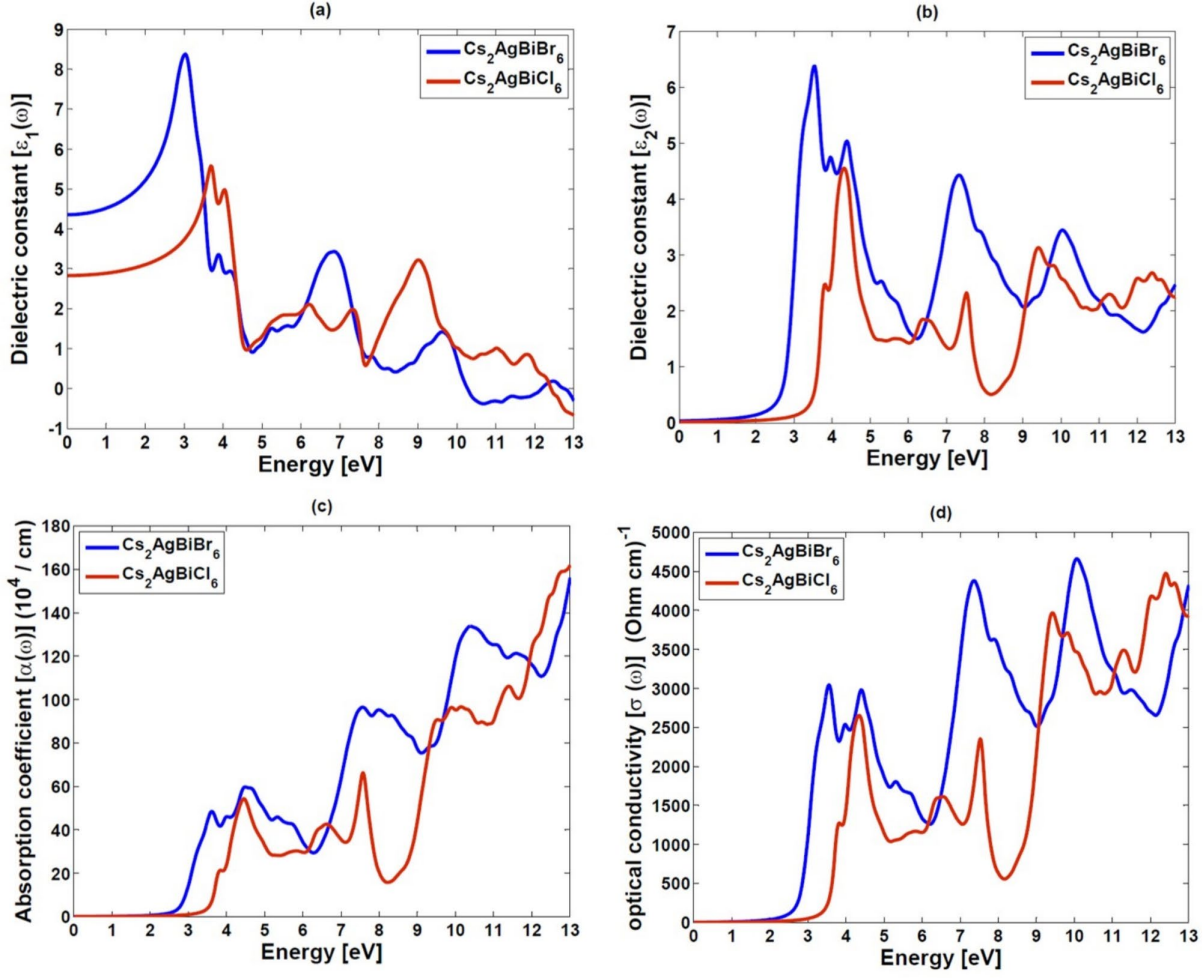
Variation of (**a**) real dielectric constant $[\varepsilon _{1}(\omega )]$, (**b**) imaginary dielectric constant $[\varepsilon _{2}(\omega )]$, (**c**) absorption coefficient $[\alpha (\omega )]$, and (**d**) optical conductivity $[\sigma (\omega )]$ with a photon energy of $Cs_{2}AgBiX_{6}$ (X = Br, Cl) compounds.

The findings of a detailed examination of the optical properties of the stable structure $Cs_{2}AgBiX_{6}$ (X = Br, Cl) as a function of incident photon energy are shown in Fig. 8(a-d). Figure 8(a) shows how $\varepsilon _{1}(\omega )$ varies for $Cs_{2}AgBiBr_{6}$ and $Cs_{2}AgBiCl_{6}$ with incident photon energy.

The sensitivity of materials with low-frequency electromagnetic radiation is shown by the calculated static dielectric constants $\varepsilon _{1}(0)$, such as 2.83 for $Cs_{2}AgBiCl_{6}$ and 4.35 for $Cs_{2}AgBiBr_{6}$. For $Cs_{2}AgBiBr_{6}$, the real part $\varepsilon _{1}(\omega )$ increases with photon energy, reaching a maximum value at approximately 3.04 eV, followed by a decline and a transition to negative values beyond 10.27 eV. In contrast, $Cs_{2}AgBiCl_{6}$ exhibits its peak $\varepsilon _{1}(\omega )$ at 3.68 eV, with negative values occurring beyond 12.42 eV, eventually showing a slight resurgence toward zero. The occurrence of negative $\varepsilon _{1}(\omega )$ values suggests conductive behavior, which can be advantageous for applications such as superlenses, optical fibers, optical filters, and electromagnetic shielding devices. This characteristic offers $Cs_{2}AgBiX_{6}$ (X = Br, Cl) an attractive material for optoelectronic applications. The imaginary component of the dielectric function, $\varepsilon _{2}(\omega )$, exhibits significant peaks in the visible and ultraviolet (UV) regions of the electromagnetic spectrum, indicating high light absorption at these wavelengths. The combined high dielectric response, light absorption, and conductive nature position $Cs_{2}AgBiX_{6}$ (X = Br, Cl) as a versatile candidate for emerging optoelectronic technologies in photodetectors.^45,46^

In Fig. 8(b), the imaginary component of the complex dielectric function, $\varepsilon _2(\omega )$, is presented for $Cs_{2}AgBiX_{6}$ compounds (X = Br, Cl). The function $\varepsilon _2(\omega )$ is essential for analyzing electronic transitions between the occupied valence band and the unoccupied conduction band, providing valuable insights into the materials’ interaction with incident electromagnetic radiation. For $Cs_{2}AgBiBr_{6}$, $\varepsilon _2(\omega )$ shows a significant electronic transition at 3.55 eV and a further significant peak appears at 5.40 eV. Similarly, for $Cs_{2}AgBiCl_{6}$, $\varepsilon _2(\omega )$ shows a high peak at 4.34 eV and another significant peak at 9.43 eV. Beyond this, the decline in $\varepsilon _2(\omega )$ at higher photon energies indicates a reduced probability of electronic transitions with increasing photon energy.

**Fig. 9:**
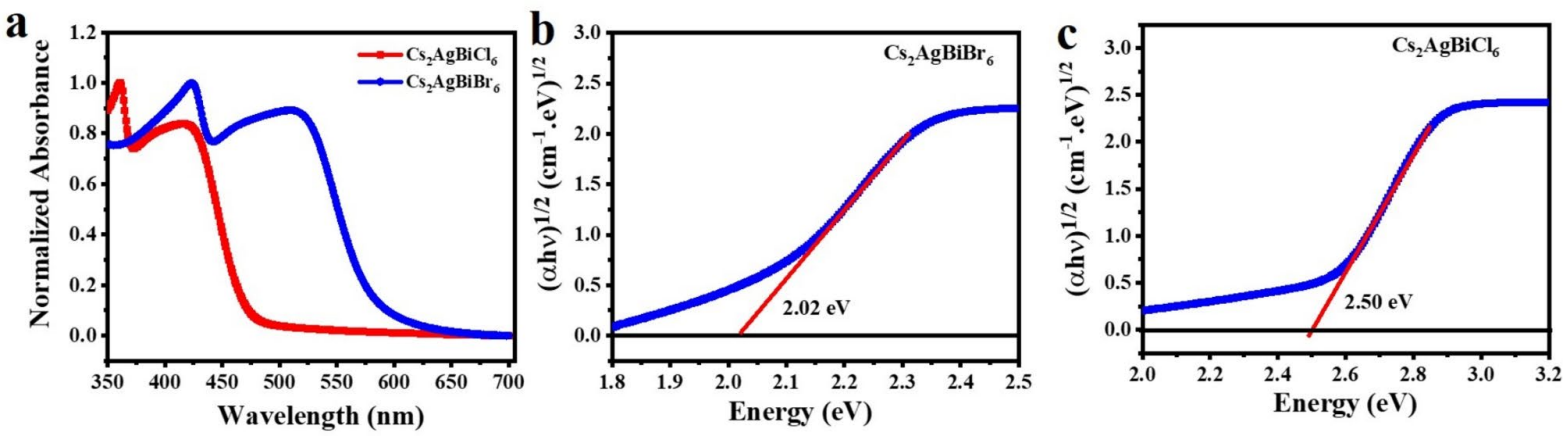
(**a**) Absorption spectra of $Cs_{2}AgBiX_{6}$ (X=Br,Cl). Calculated indirect bandgap values using the Tauc plot method for (**b**) $Cs_{2}AgBiBr_{6}$ and (**c**) $Cs_{2}AgBiCl_{6}$ systems.

These peaks observed in $\varepsilon _2(\omega )$ show the energy ranges where electronic transitions are most probable, thereby clearly showing the absorption behavior of the material. In particular, the $Cs_2AgBiX_6$ compounds (where X = Br, Cl) show significant absorption in the visible and ultraviolet (UV) spectrum, which makes them appealing choices for optoelectronic applications such as light-absorbing layers in photodetectors and photovoltaic devices. This investigation of $\varepsilon _2(\omega )$ provides significant details on the dynamics of electronic transitions and the absorption behavior of these materials, which is essential to the advancement of optical and photonic technologies.

We can utilize the dielectric constant to calculate the other optical characteristics, such as the absorption coefficient $\alpha (\omega )$, energy loss function $L(\omega )$, refractive index $n(\omega )$, optical conductivity $\sigma (\omega )$, optical reflectivity $R(\omega )$, and extinction coefficient $k(\omega )$. This interdependent approach reveals that for $Cs_2AgBiBr_6$and $Cs_2AgBiCl_6$ the absorption coefficient starts at about 1.8 eV and 2.4 eV, respectively, with a peak around 13 eV. As a result of the interaction between photons and electrons, electrons are excited from the valence band to the conduction band. These transitions account for high absorption abilities of compounds and low energy losses, making them suitable for optoelectronic applications. Furthermore, the extinction coefficient $k(\omega )$ shows in Figure $S_{2}$(b), which measures the attenuation of the input light, is closely related to the real component of the dielectric constant, supporting the close connection between light absorption and electronic structure. Optical conductivity $\sigma (\omega )$, provides valuable insight into how free carriers are generated in a material. In the case of the double perovskite compounds $Cs_2AgBiBr_6$ and $Cs_2AgBiCl_6$ shows distinct peaks in their optical conductivity spectra 4659 $(\Omega \bullet cm)^{-1}$ at 10.08 eV for $Cs_2AgBiBr_6$ and 4467 $(\Omega \bullet cm)^{-1}$ at 12.42 eV for $Cs_2AgBiCl_6$. These peaks show that as light is absorbed, its energy is transferred to the electrons, causing the chemical bonds in the material to break. Stronger light absorption leads to more bond breakage, which in turn releases more free carriers, directly enhancing the optical conductivity. This interdependent relationship between light absorption, bond disruption, and free carrier release is crucial for understanding the optoelectronic properties of these compounds and can guide future applications in devices that depend on efficient charge transport.

The refractive index $n(\omega )$ is important for understanding how these materials interact with light since it directly reflects their dielectric functions. Figure $S_{2}$(a) shows that $Cs_2AgBiBr_6$ has a higher static refractive index of 2.09 compared to 1.68 for $Cs_2AgBiCl_6$. This indicates that the Br-containing compound interacts more strongly with incoming light. This behavior is strongly related to the optical reflectivity $R(\omega )$, which, as shown in Figure $S_{2}$(c), defines the ratio of reflected to incoming energy. The static reflectivity values *R*(0) of 0.12 for $Cs_2AgBiBr_6$ and 0.06 for $Cs_2AgBiCl_6$, along with the observation that the maximum reflectivity occurs in the visible region for $Cs_2AgBiBr_6$ and in the far-ultraviolet region for $Cs_2AgBiCl_6$, suggest that these optical properties are interdependent. When combined, the refractive index and reflectivity provide a thorough understanding of the material’s optical properties, resulting in them appealing possibilities for use in UV shielding, photovoltaics, and optoelectronics. The energy loss function, $L(\omega )$, measures the energy dissipation of electrons moving within a material and plays a crucial role in understanding plasma resonances. Figure $S_{2}$(d) shows the energy loss spectra as a function of the incoming photon energy. The peaks observed in $L(\omega )$ correspond to the plasma resonance frequency $(\omega _{p})$ of the valence electrons. Notably, these peaks intensify significantly as the incoming photon energy exceeds 4 eV. These findings highlight the material’s potential for use in advanced optical systems, where high-energy plasma oscillations are critical for technological applications such as plasmonic and photonic devices.

We determined the absorbance spectra and observed the diffuse reflectance spectra to examine the optical characteristics of these single crystals. Figure 9 displays the steady-state absorption spectra for these single crystals. The absorption edge in the $Cs_{2}AgBiCl_{6}$ SC is located near 490 nm (Fig. 9a), while in the $Cs_{2}AgBiBr_{6}$ SC, it is located at 620 nm (Fig. 9a). The following relation provides the absorption coefficient^47^:

5 $$\begin{aligned} \alpha =\frac{A\left( h\nu -E_{g} \right) ^{m}}{h\nu } \end{aligned}$$

where A is known as absorbance, $h\nu$ is referred as photon energy calculated in eV units (1240/(incident wavelength in nm)). We determined the indirect band gap using the Tauc plot and estimated it as 2.02 eV for the $Cs_{2}AgBiBr_{6}$ crystal (Fig. 9b) and 2.50 eV for the $Cs_{2}AgBiCl_{6}$ crystal (Fig. 9c), which is lower than the reported band gap of the $MAPbCl_{3}$ and $MAPbBr_{3}$ single crystals^48^. We found that the cubic crystal structure and close packing are unaffected by the transition of halide ions from $Br^{-}$ to $Cl^{-}$. However, the compression of $[AgCl_{6}]^{-5}$ and $[BiCl_{6}]^{-3}$ octahedrons causes crystal distortion, which causes the bandgap to increase and energy levels to shift in $Cs_{2}AgBiCl_{6}$^49^.

**Fig. 10:**
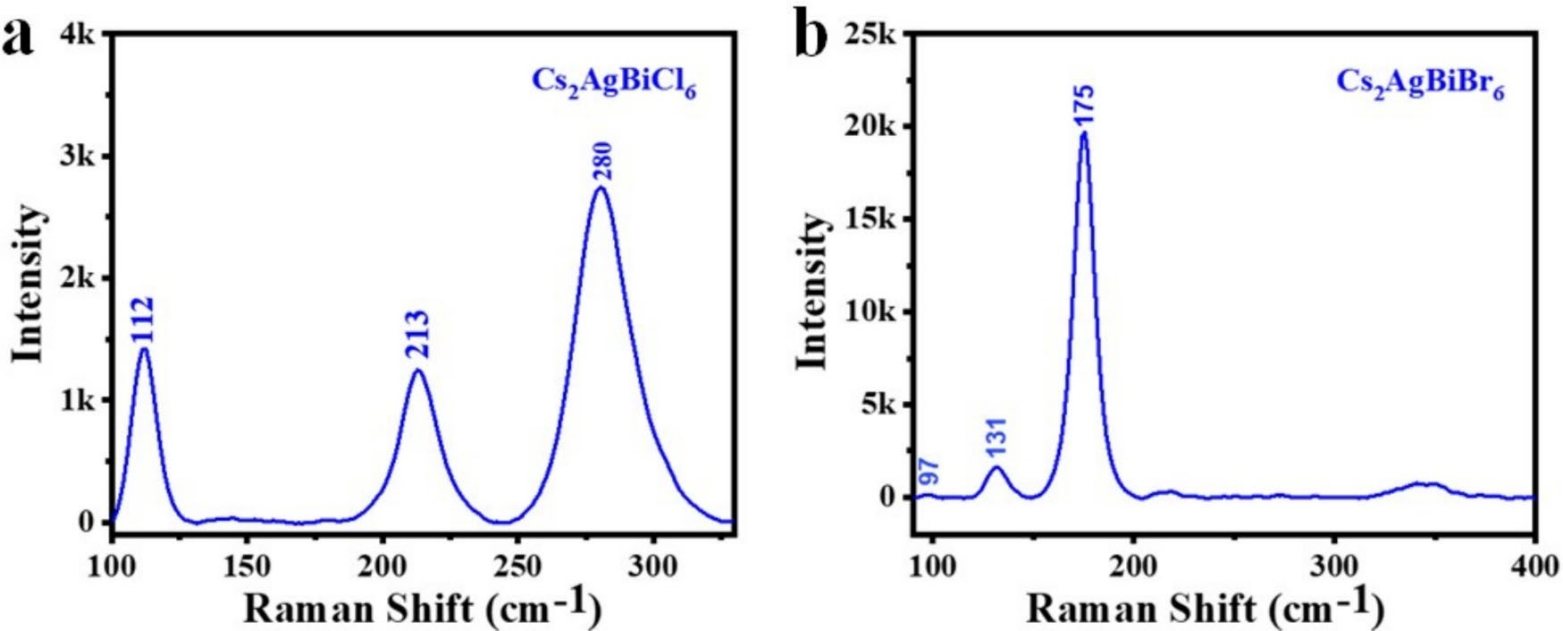
Raman spectra of (**a**) $Cs_{2}AgBiCl_{6}$ and (**b**) $Cs_{2}AgBiBr_{6}$ system.

We utilized excitation source having wavelength of 532 nm to examine Raman spectra of crystals $Cs_{2}AgBiCl_{6}$ and $Cs_{2}AgBiBr_{6}$. In the $Cs_{2}AgBiCl_{6}$ crystal, we detected the Raman peaks at 112 $cm^{-1}$, 213 $cm^{-1}$, and 280 $cm^{-1}$ (Fig. 10a). Whereas the two bands at 213 and 280 $cm^{-1}$ are associated with the stretching vibrations of the $AgCl_{6}$ octahedron with distinct vibrational symmetry of $E_{g}$ and $A_{1g}$, respectively, the band at 112 $cm^{-1}$ is attributed to a breathing vibration of the Ag-Cl bonds with $A_{2g}$ symmetry. We detected the Raman peaks at 97, 131, and 175 $cm^{-1}$ in the $Cs_{2}AgBiBr_{6}$ crystal (Fig. 10b). The peak observed at 97 $cm^{-1}$ is associated with the $A_{2g}$ (breathing) mode. As the ionic radius of Ag is larger than that of Bi, bonding deformations arise due to the large mismatch of these two sublattices. This coupled motion of Cs and Br atoms scissoring is involved in the $A_{2g}$ mode and may be related to disorders in the Ag and Bi cation configuration or a non-fully relaxed crystal structure^50^. It is determined that the asymmetric stretching vibrations of Br surrounding Bi atoms are responsible for the mode at 131 $cm^{-1}$ ($E_{g}$). The 175 $cm^{-1}$ mode develops as a result of $A_{1g}$ symmetry and is primarily composed of coordinated motion of Br atoms (symmetric bending of the Bi-Br and Ag-Br bonds within the octahedral cages). Besides these modes, we also noticed a broad peak at 348 $cm^{-1}$ (multi-photon peaks), which is equivalent to the $A_{1g}$ mode’s second-order at 175 $cm^{-1}$. If we see at the Raman spectra of both crystals, the bromides are shifted to lower energy (i.e., bromide is heavier than chloride). For both crystals, the peaks are highly symmetric, and $Cs_{2}AgBiCl_{6}$ has somewhat broader peaks than the bromide analogue. According to the literature, Ag-X type bonds have a substantially larger bonding strength than Bi-X bonds, allowing them to be regarded as discrete entities (0 D electronic dimensionality)^17^. The compelling optical properties of $Cs_{2}AgBiX_{6}$ (X = Br, Cl) make these materials ideal for high-performance photodetectors^51^ . Their strong absorption coefficients in the visible-to-UV range (peaking at $\sim 13$ eV) enable efficient light detection across broad wavelengths, critical for applications like environmental sensing, imaging, and optical communications. The high optical conductivity ($>4000 (\Omega cm)^{-1}$) ensures rapid carrier generation and fast response times, while the tunable indirect bandgaps (2.02-2.50 eV) suppress recombination, enhancing sensitivity^52^ . The cubic structure and stable Raman-active modes further ensure robustness under operational stress. Detection ranges can be improved by changing the halide composition (Br/Cl), with Br used for visible-light imaging^51^ and Cl for UV-sensitive applications^46^.

## Conclusion

The structural, electrical, and optical characteristics of lead-free double perovskites, $Cs_{2}AgBiBr_{6}$ and $Cs_{2}AgBiCl_{6}$, synthesized using a solution-processed slow-cooling approach, are extensively investigated in this study. Structural characterization results confirmed their cubic crystalline phases, whereas optical measurements demonstrated that the halide substitution successfully tunes the bandgap, with $Cs_{2}AgBiBr_{6}$ revealing a narrower bandgap of $\sim 2.02$ eV compared to the wider $\sim 2.50$ eV bandgap of $Cs_{2}AgBiCl_{6}$. X-ray photoelectron spectroscopy presented significant information about the composition of elements and binding energies, emphasizing small modifications influenced by the halide ions. Additionally, DFT calculations reinforced the presence of indirect bandgaps, aligning with the observed optical data. The results highlight how lead-free double perovskites’ optoelectronic characteristics may be optimized through halide modification, which makes these materials promising for use in photovoltaics and optoelectronics.The lower bandgap of $Cs_{2}AgBiBr_{6}$ indicates that it is suitable for visible-light absorption, whereas the broader bandgap of $Cs_{2}AgBiCl_{6}$ could prove advantageous for tandem solar cells or photodetector applications. With the goal to improve charge transfer and lower recombination losses, future studies can expand on this work by investigating other halide substitutions, alloying techniques, and interface designing. Additionally, fabricating and testing prototype devices, such as solar cells or X-ray detectors, will be crucial to fully assess the practical viability of these lead-free perovskites in sustainable optoelectronic technologies.

## Supplementary Information

Supplementary Information.

## Data Availability

The datasets generated and/or analysed during this research work would be available from corresponding author on reasonable request.
