# Supplementary material for: Impact of halide variation on the optoelectronic properties of double perovskites
Source: Sci Rep. 2025 Sep 29;15:33629. doi: 10.1038/s41598-025-98686-6 (PMC12480852; doi:10.1038/s41598-025-98686-6)
Supplement: Supplementary file 1 — Supplementary Information. [file 41598_2025_98686_MOESM1_ESM.pdf]

Impact of Halide Variation on the Optoelectronic Properties of Double Perovskites

Deepak Choudhary, Mandeep Kaur, Govind Sharma, Rahul Palsaniya, Swarnkesh Loyalka, Satpal Singh, Updesh Verma, Prashant Yadav*, and Manendra*

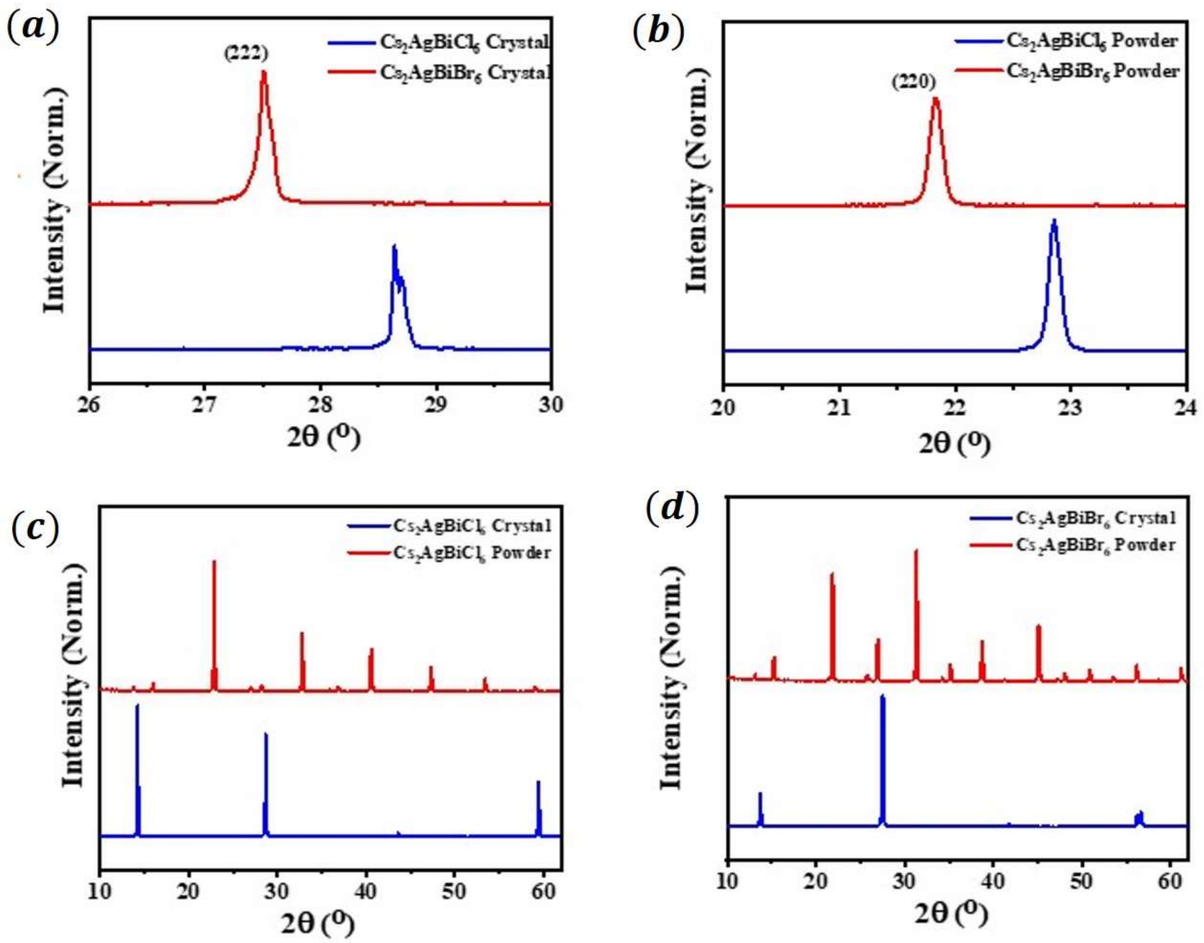

Figure S1. (a) Zoomed plot of XRD pattern of Cs_2AgBiX_6 (X = Br, Cl) crystal. (b) Zoomed plot of XRD pattern of Cs_2AgBiX_6 (X = Br, Cl) powder. Comparison of XRD patterns of powder and crystal of (c) $Cs_2AgBiCl_6$ and (d) $Cs_2AgBiBr_6$ system.

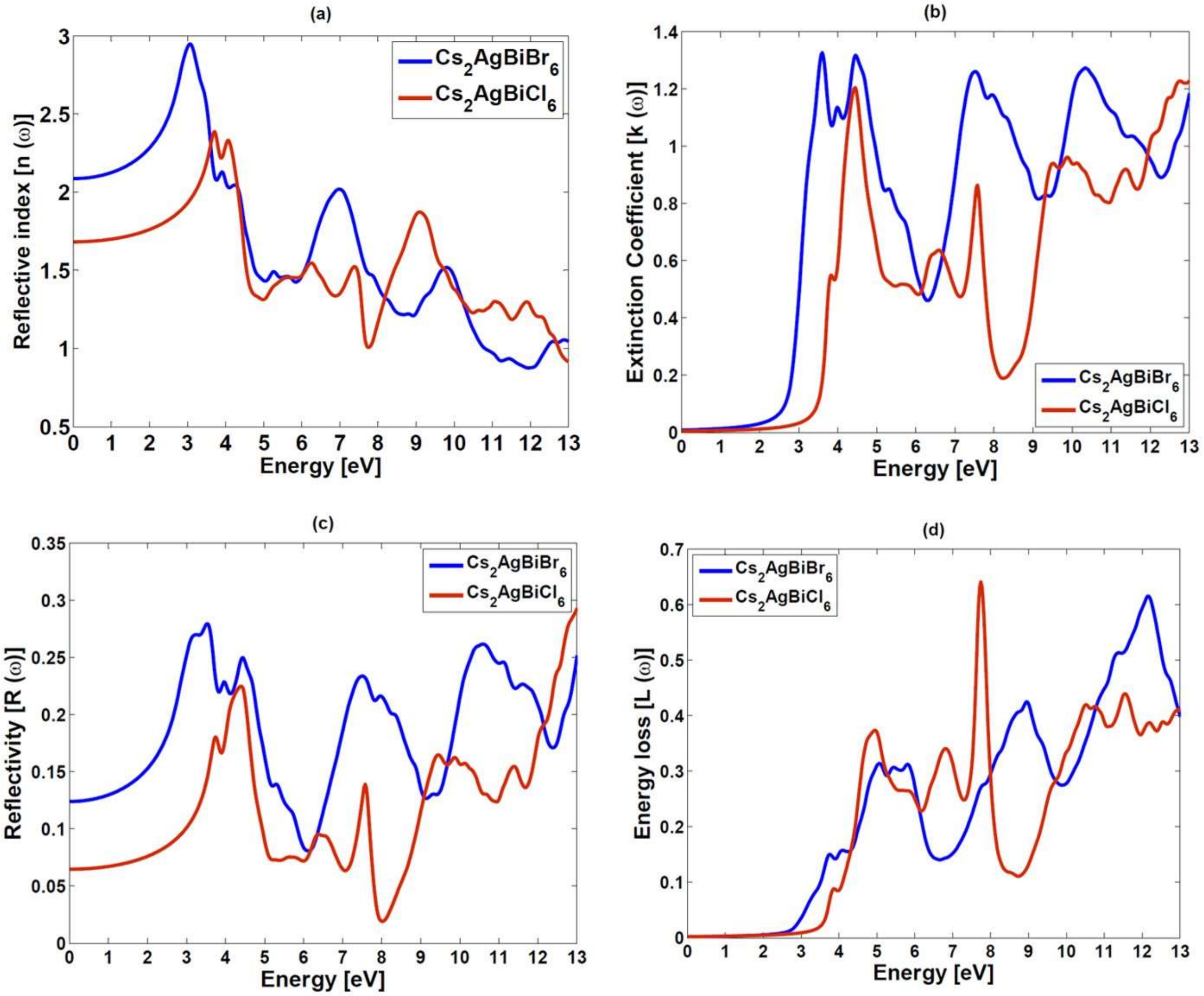

Figure S2. Variation of (a) Refractive index $n(\boldsymbol{\omega})$, (b) Extinction coefficient $k(\boldsymbol{\omega})$, (c) Reflectivity $R(\boldsymbol{\omega})$, and (d) energy-loss function $L(\omega)$ with a photon energy of compounds Cs_2AgBiX_6 (X = Br, Cl).
